# An Online Resource of Digital Stories About Cancer Genetics: Qualitative Study of Patient Preferences and Information Needs

**DOI:** 10.2196/jmir.1735

**Published:** 2011-09-30

**Authors:** Rachel Iredale, Lisa Mundy, Jennifer Hilgart

**Affiliations:** ^1^Institute of Medical GeneticsCardiff UniversityCardiffUnited Kingdom

**Keywords:** Consumer health information, familial cancer, Internet, narrative medicine, patient involvement.

## Abstract

**Background:**

The Cancer Genetics Service for Wales (CGSW) was established in 1998 as an all-Wales service for individuals with concerns about their family history of cancer. CGSW offers a range of services such as risk assessment, genetic counseling, and genetic testing. Individuals referred to cancer genetics services often have unmet information and support needs, and they value access to practical and experiential information from other patients and health professionals. As a result of the lifelong nature of genetic conditions, a fundamental challenge is to meet the ongoing needs of these patients by providing easily accessible and reliable information.

**Objectives:**

Our aims were to explore how the long-term information and support needs of CGSW patients could be met and to assess whether an online bank of digital stories about cancer genetics would be acceptable to patients.

**Methods:**

In 2009, CGSW organized patient panels across Wales. During these events, 169 patients were asked for their feedback about a potential online resource of digital stories from CGSW patients and staff. A total of 75 patients registered to take part in the project and 23 people from across Wales agreed to share their story. All participants took part in a follow-up interview.

**Results:**

Patient preferences for an online collection of cancer genetics stories were collected at the patient panels. Key topics to be covered by the stories were identified, and this feedback informed the development of the website to ensure that patients’ needs would be met. The 23 patient storytellers were aged between 28 and 75 years, and 19 were female. The digital stories reflect patients’ experiences within CGSW and the implications of living with or at risk of cancer. Follow-up interviews with patient storytellers showed that they shared their experiences as a means of helping other patients and to increase understanding of the cancer genetics service. Digital stories were also collected from 12 members of staff working at CGSW. The digital stories provide reliable and easily accessible information about cancer genetics and are hosted on the *StoryBank* website (www.cancergeneticsstorybank.co.uk).

**Conclusions:**

The Internet is one mechanism through which the long-term information and support needs of cancer genetics patients can be met. The *StoryBank* is one of the first places where patient and staff stories have been allied to every aspect of a patient pathway through a service and provides patients with an experiential perspective of the cancer genetics “journey.” The *StoryBank* was developed in direct response to patient feedback and is an innovative example of patient involvement in service development. The stories are a useful resource for newly referred patients, current patients, the general public, and health care professionals.

## Introduction

### Health Information on the Internet

Internet use is growing rapidly throughout the world and individuals are increasingly turning to the Internet as a source of health information [1]. Indeed, health websites are some of the most viewed sites on the Internet [2]. In the United Kingdom, the National Health Service (NHS) Cancer Plan advises that the Internet should be promoted as a source of information for cancer patients [3]. It has been shown that people with cancer use the Internet for a wide range of information and support needs, throughout their illness [4,5]. For example, cancer patients use the Internet to interpret symptoms; to help understand consultations; to find out about tests and treatments; as a source of support; and to hear about the experiences of other patients [5-7]. Research suggests that many patients who use the Internet are satisfied with the information they receive and that their subsequent health care decisions are influenced by the information they gain [8,9].

Despite concerns about the accuracy of health information on the Internet [1,5,10], studies have shown that when searching the Internet, people assess the credibility of a website in several ways [10-12]. For example, participants in out study looked for the source of the website and prefer noncommercial sites, which are attached to recognized centers of excellence, such as universities or well-known health centers [12]. Overall, research suggests that the Internet is a useful resource for health information and individuals continue to use the Web to find this information. Research also shows that individuals want to hear from other people who have been through similar situations [13]. This is reflected in the increasing use of online communities and social networking sites for health purposes. Bender and colleagues identified a total of 1,090,397 Facebook users who were members of 620 breast cancer groups [14]. Cancer patients also benefit from having access to online stories from other patients, especially when the stories cover their specific information needs [15].

### The Role of Stories

Health practitioners have traditionally used didactic approaches when giving information to patients. However, there is growing recognition that using stories to transmit health information may have many benefits. Narratives have been shown to improve the processing of information by capturing attention, enhancing understanding, and facilitating recall [16]. People with serious illnesses welcome the opportunity to hear from others who have had a similar experience [12,13]. One study showed that patients valued being able to access the experiences of other patients because it gave them reassurance and provided practical information that health professionals may not have perceived to be relevant [12]. Patients reported that hearing the experiences of others who had been through a similar situation would have reduced feelings of fear and isolation during their illness [12]. Stories have also been shown to be useful when presenting information to certain population groups [16,17]. For example, African American women from low-income neighborhoods who watched a video narrative from another patient describing a mammogram showed greater confidence with the procedure and had more conversations about breast cancer with family members than did women who watched a content-equivalent informational video [16].

### The Cancer Genetics Service for Wales

**Figure 1:**
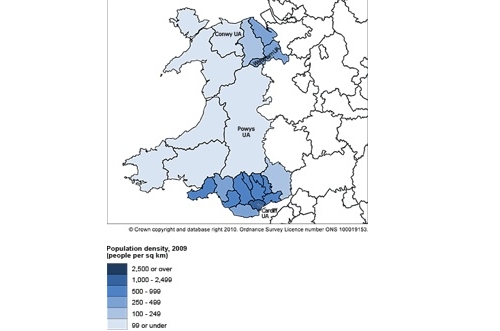
Population density map of Wales.

The population of the United Kingdom is approximately 61 million, of whom 3 million live in Wales (area approximately 8000 square miles). A majority of the Welsh population reside in South Wales (see Figure 1) [18]. The Cancer Genetics Service for Wales (CGSW) was established in 1998 for individuals with concerns about their family history of cancer. It is an all-Wales service with three sites across the country in South East Wales, South West Wales, and North Wales. To date over 26,000 people have been referred to the service. To process referrals, CGSW uses a triage system [19]. First, patients are required to complete and return a detailed family history questionnaire. This information is then used to make a cancer genetic risk assessment, with an individual classified as having an average, moderate, or high risk of inheriting cancer. Patients found to be at moderate or high risk may be offered genetic counseling, screening, and if appropriate, genetic testing. Individuals categorized as being at average risk are advised that their risk is the same as that of the general population, and in most cases they are not offered a clinic appointment. Approximately 30% of all patients referred to CGSW are classified as being at high risk [20]. Unlike at many other health care services, once a person is referred to CGSW they can remain with the service for life because of the lifelong nature of genetic conditions. In the field of genetics, most information is based on probability and uncertainty and, as a result, living with the risk of inheriting cancer is a complex experience that can affect cognitive, emotional, and social functioning [21]. Several studies have shown that individuals undergoing genetic risk assessments show increased levels of psychological distress [22,23].

### Identifying the Need for the Cancer Genetics StoryBank

It should be recognized that the information needs of cancer genetics patients are diverse and can change over time [24-27]. Individuals referred to CGSW often have not been affected with cancer themselves but have a history of cancer in their families. For these people, unlike those who have a cancer diagnosis, their status is ambiguous because they maybe at increased risk of cancer but they do not have that illness now [28]. Furthermore, these individuals may have children who might also be at increased risk of hereditary cancer. The combination of these factors can make it difficult for families involved with cancer genetics services to make sense of their situation. It is imperative to meet patients’ varying information needs because improved patient understanding may increase uptake of recommended screening and preventive measures, as well as help an individual cope with the risk assessment process, their risk categorization, and living with the risk of cancer [29,30]. 

Previous studies have found that the information needs of many patients living with a genetic risk of cancer are not being met [20,24,29] and that individuals undergoing genetic risk assessments have poor knowledge of genetics and the processes involved [30-33]. Many resources offer Web-based support to individuals with cancer, including online communities on UK cancer charity websites such as Macmillan and Breast Cancer Care. However, there are limited resources for individuals living at risk of cancer. One study examining the needs of patients referred to CGSW found that patients wanted improved access to information [20]. For example, patients sought general information about services, new developments in genetics, and details of other charities and organizations that could provide more information. Patients also wanted specific information, such as descriptions of a clinic consultation and the procedures involved in a DNA test. Patients suggested this information could be provided on a public website [20]. Another study exploring the information needs of women carrying a *BRCA* *1/* *2* gene mutation, which increases their risk of breast and ovarian cancer, found that participants felt that they and their families would benefit from an ongoing support network, which incorporated some Internet-based support [34]. Previous research with CGSW patients has shown that patients want up-to-date and accurate information about cancer genetics, to be reminded that they are never discharged from the service, and the opportunity to hear from other patients [20,35]. The *StoryBank* was therefore developed within CGSW in response to patient feedback, to address the demand for a Web-based resource for cancer genetics patients, which would meet their information and support needs on a long-term basis. 

### Involving Patients in Service Development

Patient involvement in health service development is imperative because in most Western countries there is increasing recognition that involving patients in health care is important for improving the quality of care provided [36,37]. In England and Wales health policies over the last 10 years have aimed to increase patient involvement in the NHS [38,39]. In relation to cancer, the Calman-Hine report and the NHS Cancer Plan have long emphasized that cancer services should be more patient centered and should be developed with consideration of patient views [3,40]. 

This paper describes how patients were involved in the development of the *StoryBank* [41], a website hosting a collection of digital stories from CGSW patients and staff about the cancer genetics service and associated clinical and psychosocial issues. Digital stories are the fusion of a narrative, in which the individual tells his or her story, with a series of images to illustrate the story. The aim of the *StoryBank* is to provide information and support to current and future patients, in a form that is easily accessible, reliable, engaging, and perhaps most important, allows patients to hear about the experiences of other patients. Patients have been involved at every stage of the project, from the initial conception to the launch of the website.

## Methods

### Patient Panels

The *StoryBank* has evolved as part of a 5-year program of work focusing on increasing patient involvement in service improvement and delivery at CGSW [35]. In June 2009, a newsletter was issued inviting CGSW patients to attend a patient panel. The newsletter was sent to 5906 patients, who had expressly asked to be kept informed about new initiatives. Of these patients, 28.46% (1681/5906 patients) returned the invitation, with 25.3% (425/1681 patients) expressing an interest in attending a patient panel. In total three patient panels were held across Wales during autumn 2009, with 169 participants attending (South East Wales n = 83; South West Wales n = 43; North Wales n = 43). The aims of the patient panels were for patients to decide which issues should be covered by the stories and how the Web-based resource should be developed. At the patient panels, patients were shown examples of digital stories. In groups of 8–10, patients were asked to discuss how digital stories could best meet CGSW patients’ information and support needs and their preferences regarding how they would like to tell their stories. Each group was given a feedback sheet to record their comments. All the feedback sheets were collected at the end of each patient panel. We collected 359 comments, 111 of which were excluded as they related to comments about the digital stories that were shown or about the patient panel itself. Therefore, we analyzed the content of 248 comments about patient preferences for the *StoryBank* [42]. After detailed review of the data, two authors (RI and JH) developed the standardized coding categories. RI and JH then independently coded the comments, with any discrepancies resolved through discussion. Patient preferences for the digital stories were examined by calculating simple frequencies on all coding categories. Information obtained during this task was used to plan the *StoryBank* project, and patients were also invited to register to share their own cancer genetics story.

### Participants

In total, 75 patients at the patient panels said they would like to take part in the *StoryBank*. In spring 2010, further information about the project and a consent form were sent to these patients. Patients were asked to return the consent form to confirm they wanted to share their story and were happy to be contacted. From across Wales, 24 patients returned a consent form, although one individual did not progress past the phone call stage. The 23 patients who shared their story ranged in age from 28 to 75 years and 19 were female; 12 (52%) were classified as being at high risk, 6 (26%) as moderate risk, and 3 (13%) as average risk; 2 (9%) were relatives of patients who had been referred to the service.

### Procedure

Once a consent form had been received, each participant was contacted by telephone to arrange a convenient date and location to record their story. The majority of participants (20/23, 87%) chose to record their story at home. 

During the appointments, which took the form of an unstructured interview, participants were asked to describe their experience of the cancer genetics service. The researchers’ role was simply to facilitate the telling of the story and to ask patients for more information when clarification was needed. All recordings were made using a high-quality audio recorder. The audio recordings lasted between 17 and 60 minutes. Participants were asked to provide photographs to illustrate their story, if they desired. Only one patient did not want to use personal photographs and another patient wished to remain anonymous and so provided photographs that did not include family members or friends. 

Stories were also collected from 12 members of staff working in, or with, CGSW. Staff included specialists in clinical genetics, a family history coordinator, and a clinical scientist at the All Wales Molecular Genetics Laboratory. Staff provided factual stories about cancer, genetics, CGSW, and the processes involved. Recordings of staff stories lasted between 10 and 45 minutes.

All interviews were transcribed verbatim. Both patient and staff stories were edited to short clips, between 40 and 300 seconds in duration. More than 1 story was edited for 12 of the storytellers. All stories were seen and verified by every storyteller before being uploaded onto the website. The 53 stories from patients and staff are hosted on Vimeo (Vimeo, LLC, New York, NY, USA), a video hosting website, and the stories are embedded within the *StoryBank* website [41].

### Evaluation

The *StoryBank* was evaluated on two levels: first, a brief telephone follow-up interview was conducted with all 23 patient storytellers approximately 2 weeks after the storytelling appointment, to explore their reasons for taking part in the *StoryBank* and their experiences of telling their story. All interviews were recorded and transcribed verbatim. The content of the follow-up interview was analyzed to summarize patients’ evaluation of sharing their story and their views about the *StoryBank* [42]. Each transcript was read carefully and all relevant text was highlighted. All highlighted text from three to four transcripts was used to develop preliminary categories. The remaining transcripts were coded using these categories and by adding new categories where data did not fit into an existing category [43]. The data in all the categories were then examined independently by two authors (RI and JH), which led to some categories being combined. This resulted in three main categories of storytellers’ reasons for contributing to the *StoryBank* and how they hoped their stories would benefit website users. For the second level of evaluation, a brief online survey was incorporated into the website to capture data about who was using the website and to give users the opportunity to leave feedback about the site. As this evaluation is ongoing, data from the online survey will be presented elsewhere.

## Results

### Patient Preferences for the StoryBank

At the patient panels, patients suggested topics that should be included in the *StoryBank*. These included tracing family histories; living with the risk of cancer; and telling family members, such as children, about their risk of inherited cancer. On the feedback forms, patients commented that the stories should convey personal experiences and practical information about the service and cancer genetics. Patients also expressed a preference for the *StoryBank* to include stories about a range of cancers and different levels of risk. Patient comments about the *StoryBank* and how they were used to inform the development of the website are shown in Table 1. Feedback about the project at the patient panels was generally positive with comments such as *“I would have liked to have seen a digital story when I was going through the journey, for a better understanding”* and *“a great idea and a valuable service”*.

**Table 1:** Patient preferences for the StoryBank (N = 248) and how they have been considered during website development

Coding category	Patient comments	Number of comments	% of total comments (N = 248)	How suggestions informed website development
Stories about personal experiences	*A good digital story would be how a patient dealt with their journey through the genetic history process.*	74	30	23 patient stories were collected and hosted on the website about their experiences of cancer and cancer genetics
Stories providing service information	*Initial digital stories need to be informative about the service including contact information.*	69	28	12 staff stories containing factual information about the service were collected and hosted on the website
The importance of including a variety of stories (eg, different age groups, different cancers)	*Variety of stories, ages, backgrounds.Different stories for different cancers.*	28	11	Male and female storytellers are a range of ages, affected by different cancers and at various levels of risk
Preference for positive stories	*In general stories need to be more positive. People don’t want to hear the bad, they want encouragement and support.*	26	11	Many of the stories hosted on the website relate to the benefits of being referred to the cancer genetics service
Stories about the impact of genetics on families	*What it entails for partners of people affected.*	13	5	Storytellers shared their personal experiences of cancer genetics and the impact this had on their families, especially their children
Information about other support services	*Relevant information about various different counseling bodies available from Samaritans to grief counselors*	12	5	The website contains links to external sources of support including charities such as Tenovus, the cancer charity in Wales.
Stories from family members of patients	*Show the whole family’s feelings, including older and younger members.*	10	4	2 storytellers are related to CGSW patients and their stories are about the impact upon them
Would like a discussion forum	*Forum to discuss details on genetics—knowing you have a faulty gene which has yet to be identified and how you cope with it.*	2	1	A discussion forum is not currently feasible, although users of the website are encouraged to contact CGSW if they have any queries or concerns
Other comments (eg, relating to own experience of cancer)		14	6	Not applicable

### Developing the Website

A total of 53 individual stories about the various aspects of the cancer genetics service were produced. Of these, 37 clips are from patients about their personal experiences and 16 clips are from staff providing more practical information about the service (see Multimedia Appendix 1). The content of the *StoryBank* was developed using the information provided directly from service users at the patient panels. First, the routine patient journey through CGSW was identified and divided into key topics. These topics were as follows:

- What is the Cancer Genetics Service for Wales?
- Completing the family history questionnaire
- Waiting for the results of a risk assessment
- Being told if you are at low, moderate, or high risk of cancer
- Attending genetic counseling
- Having a genetic test
- Making decisions about screening and surgery
- Living with cancer
- Information about rare cancers
- Sources of support.

**Figure 2:**
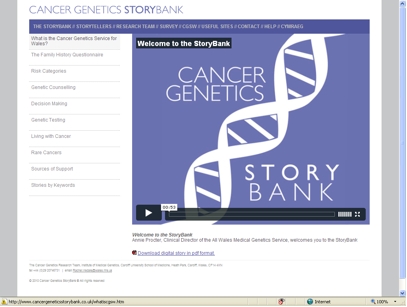
Screenshot from the *StoryBank* (www.cancergeneticsstorybank.co.uk).

**Figure 3:**
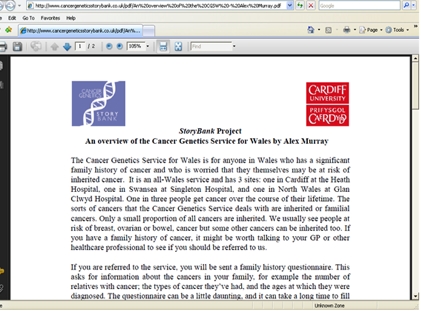
Screenshot of PDF story transcript from the *StoryBank* (www.cancergeneticsstorybank.co.uk).

Next, the main themes of each patient and staff story were identified and mapped onto the key topics. The stories are presented on the website under these key topics (see Figure 2). However, there is also a page on the site that lists all the storytellers and their stories, so that users can also search for stories via the storyteller in order to see the journey one particular person might have gone through [41]. All the stories were transcribed and the transcripts are available to download from the website as PDF files (see Figure 3 and Multimedia Appendix 2).

The website was designed with the aid of a professional Web designer, and patients and staff at CGSW were invited to give their feedback about the website before it was launched. A special event was held to mark the launch of the website on November 25, 2010. The event was opened by the First Minister of Wales, Rt Hon Carwyn Jones AM, and was attended by 105 guests, including the storytellers and their families; staff at CGSW; and others interested in cancer, genetics, and promoting patient-centered service delivery across NHS Wales. In the first month (November 25, 2010 to December 25, 2010) since the website was launched, the stories were viewed 494 times and were played in 15 countries across the world, including the USA, Australia, South Korea, the Netherlands, and Spain.

### Evaluation

Data from the follow-up interviews demonstrate that the storytellers were all positive about the *StoryBank* and the experience of sharing their story. Table 2 shows the three coding categories that were developed from the content analysis of the interview transcripts. A majority of the storytellers (19/23, 83%) stated that they wanted to share their story as a means of helping others who were going through a similar experience. For example, one patient said

I’d done a lot of Internet searching and I couldn’t find a story similar to mine. So I thought if I get an opportunity to say my story then I will,because at some point there will be somebody going through the same thing as me.

Another patient explained

I’ve got first hand experience of what it’s like and hopefully somebody else can experience positive feelings from what I’ve said

**Table 2:** Storytellers’ reflections on sharing their story (N = 23)

Coding category	Examples	N (%)
Providing reassurance and support for other patients	*...to support people through it, to let them know there’s light at the end of the tunnel and to know they’re not on their own everybody feels that way or a lot of people feel that way.*	19 (83)
	*We have a very rare, it’s a very rare cancer and I kind of think rare cancers need to be included you know because there’s a lot written, lots of support and everything else for the common cancers and I think then that makes, well certainly made me with a rare cancer feel kind of more isolated if you like.*	
Increasing knowledge and understanding of the cancer genetics service	*When we were involved with the genetics service, we’d not known anything about it, um and we just felt that if we could do something that would help others understand what might be in front of them, then it might be worth doing.*	9 (39)
	*I think that it’s really quite important that people go with that in mind...that they understand that the test isn’t absolutely the end of the story. If you get a negative result there still might be something beyond that.*	
Reducing the fear and uncertainty surrounding cancer genetics	*Well I hope people who listen or see it will find it encouraging to go forward and seek advice.*	6 (26)
	*I just hope that through my experience and through the way I’ve tried to explain things, it makes it less of a specter and it makes it less of an unknown quantity.*	

## Discussion

During 3 years of patient-centered research at CGSW we found that patients want access to factual information about cancer genetic services and to hear personal stories from a variety of patients who are similar to themselves in terms of cancer genetic risk level, age, and gender [20,35]. Patients were invited to attend a patient panel to suggest topics to be included in the *StoryBank*, a Web-based resource of digital stories from patients and staff at CGSW. These suggestions were used to ensure that the *StoryBank* met the information and support needs of current and future patients and that it was developed in accordance with patient preferences. Increasing patient involvement in service development is vital because it leads to improved communication and decision making, and ultimately to better health outcomes [37]. We suggest that the *StoryBank* project is an innovative example of involving patients in service development and delivery.

The success of websites such as healthtalkonline (formerly DIPEx) [13,44] and the use of patient stories on UK cancer charity websites such as Breast Cancer Care and Macmillan demonstrates that patients want, and indeed use, the opportunity to listen to individuals who have had similar experiences. Incorporating stories from CGSW patients with information from staff members, and mapping these onto every key stage of the cancer genetics journey, allows us to provide information in a form that is reliable, accessible, and engaging. Providing patients with the information they require allows individuals to have a better understanding of the risk assessment and associated processes [31]. This in turn results in several benefits such as more realistic patient expectations, and ultimately helps individuals cope with their risk assessment and risk categorization [29,30]. The *StoryBank* is one of the first places where patient stories have been aligned with the patient pathway through a health service. By providing narrative accounts from patients who have already been referred to CGSW, about how they have coped with the emotional challenges they faced, we hope that future patients will gain encouragement and support as they progress on their own journey through the service. 

 The *StoryBank* also informs patients about the psychosocial aspects of genetic testing and where to access additional information and support, which are often omitted from standard information leaflets provided in genetics clinics [45]. Presenting health information in a digital format can also be beneficial for patients with low literacy levels [46], and the availability of cancer genetic information before counseling may help patients with lower literacy to prepare for their clinic appointment [47]. Patient narratives have also been shown to improve understanding and aid recall of health-related information [16,36]. The data from the follow-up telephone interviews also show that participants wanted to share their story to increase other patients’ understanding of the services provided by CGSW and to convey information about what to expect from genetic risk assessment, genetic counseling, and genetic testing.

By hosting patient and staff digital stories on a website, the *StoryBank* allows individuals to access information about CGSW and hear from other patients 24 hours a day, from anywhere in the world. This is particularly beneficial for CGSW, which, as an all-Wales service, receives referrals from patients across the country. Many people in Wales live in rural communities with poor transport links [48,49]. Thus, one of the challenges faced by CGSW was meeting the information and support needs of all its patients, while recognizing the geographical constraints of Wales. The *StoryBank* provides a solution to meeting these patient needs.

The challenge for the future is to ensure that health professionals are aware of the website and the information it provides. As individuals continue to turn to the Internet for health information, it is vital that health professionals are familiar with the information that is available and are able to direct patients to the relevant resources [50]. Staff at CGSW have been involved with the project from the patient panels to the development and launch of the website. Furthermore, all staff members with patient contact at CGSW have been asked to include the *StoryBank* website address on correspondence with patients. Our task is to continue to increase awareness of the website both within CGSW and more widely. For example, general practitioners are one of the main routes through which patients are referred to CGSW [19]. Thus, it is important that they understand the service provided and the area of cancer genetics more generally. 

### Conclusions

In conclusion, the *StoryBank* was developed in response to patient feedback to meet the long-term information and support needs of cancer genetics patients. The *StoryBank* provides a Web-based resource of reliable, accessible, and engaging information for current and future cancer genetics patients. The patients’ stories communicate an experiential perspective of the cancer genetics service. The *StoryBank* is one of the first places where patient and staff stories have been mapped onto each stage of a service, which will help people gain a sense of the journey they too may experience. We suggest that the *StoryBank* could also be useful in raising awareness of cancer genetics and CGSW among health care professionals. The *StoryBank* is an innovative online resource, developed in collaboration with current patients, which could be applied to other genetic or other long-term chronic health conditions, such as diabetes and asthma, in the NHS and elsewhere.

## Multimedia Appendix 1

Digital story (video) from the StoryBank website about the Cancer Genetics Service for Wales.

## Multimedia Appendix 2

PDF transcript of the digital story about the Cancer Genetics Service for Wales.

## Abbreviations

CGSW: Cancer Genetics Service for Wales
NHS: National Health Service
