# Supplementary material for: An Online Resource of Digital Stories About Cancer Genetics: Qualitative Study of Patient Preferences and Information Needs
Source: J Med Internet Res. 2011 Sep 30;13(3):e78. doi: 10.2196/jmir.1735 (PMC3222166; doi:10.2196/jmir.1735)
Supplement: Supplementary file 2 [file jmir_v13i3e78_app2.pdf]

***StoryBank* Project**

An overview of the Cancer Genetics Service for Wales by Alex Murray

The Cancer Genetics Service for Wales is for anyone in Wales who has a significant family history of cancer and who is worried that they themselves may be at risk of inherited cancer. It is an all-Wales service and has 3 sites: one in Cardiff at the Heath Hospital, one in Swansea at Singleton Hospital, and one in North Wales at Glan Clwyd Hospital. One in three people get cancer over the course of their lifetime. The sorts of cancers that the Cancer Genetics Service deals with are inherited or familial cancers. Only a small proportion of all cancers are inherited. We usually see people at risk of breast, ovarian or bowel, cancer but some other cancers can be inherited too. If you have a family history of cancer, it might be worth talking to your GP or other healthcare professional to see if you should be referred to us.

If you are referred to the service, you will be sent a family history questionnaire. This asks for information about the cancers in your family, for example the number of relatives with cancer; the types of cancer they've had, and the ages at which they were diagnosed. The questionnaire can be a little daunting, and it can take a long time to fill in, because so much information about the history of cancer in your family is required. We realise that it can also sometimes be upsetting thinking and talking about your family history.

When you have filled in your family history questionnaire, please return it to us as soon as you can. We understand that you might struggle to gather all the information we ask for, so don't worry if some of it's missing. We would much rather receive a partly filled out questionnaire than nothing at all, because without it we can't move on with your assessment. If the questionnaire is not returned, then we will assume you do not want to continue with your referral.

Once the questionnaire has been returned, a family history co-ordinator will check the details of your family history and draw up a family tree. This might involve calling you to gather more information and reviewing medical notes and pathology records. Once we have confirmed as much detail about your family history as we can, we will assess your risk. To do this we use our experience of other families with hereditary cancer and in some cases a computerised risk assessment software package. The risk for you and your relatives will be categorised as either average, moderate or high risk.

Average risk means your risk isn't significantly higher than anyone else in the general population. There is still some risk, because everyone is at some risk of cancer and you should adopt a healthy diet and lifestyle. Average risk patients are not usually invited for a clinic appointment. If you have been categorised as moderate risk you may be advised of your risk by letter or you may be offered a clinic appointment with a genetic counsellor or doctor. You may also be offered extra screening, such as earlier or more frequent mammograms. If you are classed as high risk, you will be offered a clinic appointment in your local cancer genetics clinic to talk about risk management options, such as screening and sometimes risk-reducing surgery. Some patients will be offered genetic testing.

Most clinic appointments in cancer genetics last about an hour. Generally there is no physical examination. Most of the time is taken up discussing the family history and the implications for you and your relatives, to enable you to make choices about how best to look after yourself. If you are offered a genetic test, a blood sample may be collected at the appointment or at a later date. You are welcome to bring a family member or friend to the appointment with you, if you wish.

Genetic testing involves sending a blood sample to the laboratory to look for genes that may be the cause of the cancers in your family. Sometimes we can find the gene responsible, sometimes we can’t. Searching for a gene can take a long time but as soon as the results are available we will let you know. We can then discuss the implications in more detail and give you further advice and support.

If anything in your family history changes after we have assessed you, such as another family member getting cancer, we would really appreciate you contacting us, as your risk may change too. The most important thing to remember about the Cancer Genetics Service for Wales is that once you have been referred and been assessed by us - whatever risk category you are in - you can contact us again directly, at any time.

For more information about the *StoryBank* please contact:

Dr Rachel Iredale, Senior Research Fellow, Institute of Medical Genetics, Cardiff University School of Medicine

email: Rachel.Iredale@wales.nhs.uk

Telephone: 02920 746731

www.cancergeneticsstorybank.co.uk
